# Comparison of *Saccharomyces cerevisiae* strains of clinical and nonclinical origin by molecular typing and determination of putative virulence traits

**DOI:** 10.1111/j.1567-1364.2008.00365.x

**Published:** 2008-03-18

**Authors:** Trine Danø Klingberg, Urska Lesnik, Nils Arneborg, Peter Raspor, Lene Jespersen

**Affiliations:** 1 Department of Food Science, Faculty of Life Sciences, University of Copenhagen Frederiksberg C, Denmark; 2 Department of Food Science and Technology, Biotechnical Faculty, University of Ljubljana Slovenia

**Keywords:** *Saccharomyces cerevisiae*, clinical strains, PFGE, Caco-2 cells, adhesion, transepithelial electrical resistance

## Abstract

*Saccharomyces cerevisiae* strains of clinical and nonclinical origin were compared by pulse field gel electrophoresis. Complete separation between strains of clinical origin and food strains by their chromosome length polymorphism was not obtained even though there was a tendency for the clinical and food strains to cluster separately. All the investigated strains, except for one food strain, were able to grow at temperatures ≥37 °C but not at 42 °C. Great strain variations were observed in pseudohyphal growth and invasiveness, but the characters were not linked to strains of clinical origin. The adhesion capacities of the yeast strains to a human intestinal epithelial cell line (Caco-2) in response to different nutritional availabilities were determined, as were the effects of the strains on the transepithelial electrical resistance (TER) across polarized monolayers of Caco-2 cells. The yeast strains displayed very low adhesion capacities to Caco-2 cells (0.6–6.2%), and no significant difference was observed between the strains of clinical and nonclinical origin. Both *S. cerevisiae* strains of clinical and non-clinical origin increased the TER of polarized monolayers of Caco-2 cells. Based on the results obtained in this study, no specific virulence factor was found that clearly separated the strains of clinical origin from the strains of nonclinical origin. On the contrary, all investigated strains of *S. cerevisiae* were found to strengthen the epithelial barrier function.

## Introduction

An increasing number of reports about the involvement of *Saccharomyces cerevisiae* in superficial and life-threatening systemic diseases (Hennequin *et al.*, 2000; Perapoch *et al.*, 2000; Lherm *et al.*, 2002; Wheeler *et al.*, 2003; Munoz *et al.*, 2005; de Llanos *et al.*, 2006a, b) have led to the replacement of this organism from the group of generally recognized as safe (GRAS) to a group of opportunistic pathogens of low virulence (de Hoog, 1996). Also, increasing evidence of infections by probiotic strains of *Saccharomyces cerevisiae* var. *boulardii* (van der Aa Kühle & Jespersen, 2003) has been reported (Perapoch *et al.*, 2000; Cassone *et al.*, 2003; Riquelme *et al.*, 2003; de Llanos *et al.*, 2006b). Clinical cases are caused by oral administration, blood infections or other ways of exogenous inoculation. The patients infected are in most cases premature children, elderly people or patients suffering from immunosuppression due to AIDS, treatment with immunosuppressive agents, etc. However, it is still unclear whether some *S. cerevisiae* strains are more likely to cause infections than others (Nyirjesy *et al.*, 1995; Perapoch *et al.*, 2000; Lherm *et al.*, 2002; Cassone *et al.*, 2003; Riquelme *et al.*, 2003).

Many epidemiological characterizations of *S. cerevisiae* isolated from clinical specimens have been performed (Zerva *et al.*, 1996; Clemons *et al.*, 1997; McCullough *et al.*, 1998; Posteraro *et al.*, 1999), but the strains are often not characterized by appropriate typing techniques. Determination of chromosome length polymorphism (CLP) is well recognized as a useful method for typing and characterization of strains of *S. cerevisiae* from different types of foods (Jespersen *et al.*, 1999, 2000, 2005; van der Aa Kühle *et al.*, 2001) and is used in the food industry for typing, tracing and characterization of starter cultures and contaminants. However, the method has not been used for characterization of *S. cerevisiae* strains of clinical origin.

Some of the reported key elements of virulence are the ability to grow at high temperatures (39–42°) and the ability of the yeast species *in vitro* to perform a dimorphic switch between unicellular growth and pseudohyphal growth, which is generally considered an important adaptive response to environmental stress conditions such as e.g. nutritional deprivation (Lorenz *et al.*, 2000; Gagiano *et al.*, 2002; Palecek *et al.*, 2002; Casalone *et al.*, 2005). Invasive growth on solid agar has also been reported as a virulent trait (McCusker *et al.*, 1994a, b; Palecek *et al.*, 2002; Casalone *et al.*, 2005).

Upon oral administration, adhesion to mammalian epithelial cells is likely to be crucial to ensure maintenance of the yeast in the human host and a prerequisite for invasiveness and clinical symptoms (Kleinschmidt *et al.*, 2005; Kumamoto & Vinces, 2005). Previously, mammalian epithelial cells have been used as *in vitro* models for the adhesion of e.g. probiotic bacteria and yeasts to the gastrointestinal (GI) tract (van der Aa Kühle *et al.*, 2005; Klingberg *et al.*, 2005a) and to determine the permeability of the epithelium by measurements of the transepithelial electrical resistance (TER) (Klingberg *et al.*, 2005b). The addition of various pathogenic bacteria has been shown to increase the permeability of the epithelium, leading to deterioration of the epithelial barrier function (Nusrat *et al.*, 2001; Tafazoli *et al.*, 2003), whereas probiotics have been shown to decrease the permeability of the epithelium (Resta-Lenert & Barrett, 2003; Klingberg *et al.*, 2005b). Measurements of the TER of polarized monolayers of epithelial cells are therefore seen as a useful method of providing information about potential virulence or the probiotic effect of microorganisms upon oral administration.

As *S. cerevisiae* is widely present in many foods and beverages, it has been the aim of the present study to investigate in *in vitro* models whether strains of *S. cerevisiae* isolated from clinical specimens could be separated from strains of *S. cerevisiae* originating from food or being used as probiotics. The strains were compared by their CLP and previously reported virulence traits. Yeast adhesion and influence on the TER of the epithelium were evaluated using a human intestinal epithelial cell line (Caco-2).

## Materials and methods

### Yeast strains and growth media

The origins of the clinical and nonclinical strains of *S. cerevisiae* used in the present study are listed in Table 1. Strains denoted SSI are isolated from clinical specimens and kindly provided by Statens Serum Institute, Copenhagen, Denmark, whereas strains denoted YJM were obtained from Centraalbureau voor Schimmelcultures (CBS), Utrecht, the Netherlands. These strains were included as they have previously been shown to display different degrees of virulence in CD-1 mice (McCusker *et al.*, 1994a, b). The probiotic strains (7103, 259 and LSB) were purchased from different commercial suppliers (see Table 1), and strains isolated from food (56, D7, A6, A18, C1, KVL012) were obtained from the yeast collection at the Department of Food Science, The University of Copenhagen, Frederiksberg, Denmark. The haploid strain S288C (CBS8803) was obtained from CBS and included as reference. All strains were maintained at −80 °C in 20% (v/v) glycerol (Merck, Darmstadt, Germany). Strains were propagated in YPD broth [1% (w/v) glucose (Merck), 1% (w/v) Bacto Peptone (Difco, Detroit, MI), 0.5% (w/v) yeast extract (Difco), pH 5.6] for 24 h at 30 °C.

**Table 1:** Strains of *Saccharomyces cerevisiae* included in the present study

Name	Origin	Supplier
Strains of clinical origin		
SSI1	Toe	Statens Serum Institute, Copenhagen Denmark*
SSI2	Adrenal gland	
SSI3	Blood	
SSI4	(RH 7806)	
SSI5	(Hvidovre112)	
SSI6	Vagina	
SSI7	Vagina	
SSI8	Vagina	
SSI9	Vagina	
YJM128 (CBS 7833)†	Lungs	Centraalbureau voor Schimmelcultures (CBS), Utrecht, the Netherlands
YJM222 (CBS 7834)†	Man	
YJM273 (CBS 7835)†	Peritoneal fluid	
YJM308 (CBS 7836)†	Paracentesis fluid	
YJM309 (CBS 7837)†	Blood	
YJM310 (CBS 7838)†	Man	
YJM311 (CBS 7839)†	Bile tub	
YJM312 (CBS 7840)†	Ascites fluid	
Probiotic strains		
7103‡,§	Ultra-Levure, batch 7103 (*S. cerevisiae* var. *boulardii*)	Laboratoires Biocodex, Montrouge, France
259‡,§	Precosa®, batch 259 (*S. cerevisiae* var. *boulardii*)	Logic aps., Lynge, Denmark
LSB‡,§	Levucell® SB, feed supplement (*S. cerevisiae* var. *boulardii*)	Euro Tier, Niederzissen, Germany
Food strains		
56§,¶	Danish blue veined cheese	The Faculty of Life Sciences, Department of Food Science, Copenhagen, Denmark
D7§	Gorgonzola, Italy	
A6§,∥	Sorghum beer, Ghana	
A18§,∥	Sorghum beer, Ghana	
C1§,∥	Sorghum beer, Burkina Faso	
KVL012§,**	Brewing ale yeast	
Laboratory haploid strain		
S288C (CBS 8803)		CBS, Utrecht, the Netherlands

* Strains of clinical origin were identified by use of API ID 32 C kit (BioMerieux SA, Marcy L'Etoile, France) according to the manufacturer's instructions. All strains were identified as *Saccharomyces cerevisiae*.

† The strains were purchased from the CBS collection. The names used in this paper are in coherence with the names used by McCusker *et al.* (1994a, b), Byron *et al.* (1995) and Clemons *et al.* (1997).

‡ *Saccharomyces cerevisiae* var. *boulardii* (van der Aa Kühle & Jespersen, 2003)

§ van der Aa Kühle *et al.* (2005).

¶ Hansen & Jakobsen (2001).

∥ van der Aa Kühle *et al.* (2001).

** Jespersen *et al.* (1999).

For induction of pseudohyphae, synthetic low-nitrogen medium [SLAD; 2% (w/v) glucose (Merck), 2% (w/v) Bacto Peptone (Difco), 0.65% (w/v) yeast nitrogen base without amino acids and ammonium sulphate (Difco) and 50 μmol L^−1^ ammonium sulphate (Merck)] (Gimeno *et al.*, 1992) and synthetic complete low dextrose medium [SCLD; 0.17% (w/v) yeast nitrogen base (Difco), 40 mmol L^−1^ ammonium sulphate (Merck), 0.1% (w/v) glucose (Merck)] (Gagiano *et al.*, 2003) were used. For preparation of agar plates, 2% (w/v) of agar (Difco) was added.

### CLP and cluster analysis

Yeast cultures were grown in YPG broth [10 g L^−1^ yeast extract (Difco), 20 g L^−1^ Bacto Peptone (Difco) and 40 g L^−1^ glucose (Merck), pH 5.6] at 25 °C for 48 h, and successively recultivated for 24 h. Spheroplasts and agar blocks were prepared as described previously by Hayford & Jespersen (1999). The gel was run at 10 °C for 40 h (90 s for 14 h, 105 s for 12 h and 120 s for 14 h) at 100–120 mA and 160 V (CHEF-DR®III System, Bio-Rad Laboratories) in TBE buffer [45 mmol L^−1^ Tris-base (Sigma), 44 mmol L^−1^ boric acid (Sigma) and 1 mmol L^−1^ EDTA]. Yeast DNA marker (Pharmacia) was used for determination of chromosome size. The gel was stained with 1 mg L^−1^ ethidium bromide (Sigma) for 30 min, washed in Milli-Q water for 5 min, visualized with a UV transilluminator (Pharmacia) and photographed with a land camera (Polaroid MPE, Cambridge, MA). The pulse-field gel electrophoresis (PFGE) analyses of the strains were repeated twice in two separate experiments. The software bionumerics 2.50 (Applied Maths, Kortrijk, Belgium) was used for normalization and processing of the yeast chromosome profiles. Cluster analysis was based on the Dice coefficient and the unweighted pair group algorithm with arithmetic averages (UPGMA).

### Growth temperature assays

YPD broth (10 mL) was inoculated with a single yeast colony, grown on YPD agar for 48 h at 30 °C. Growth was determined by visual examination following 3, 7, 14 and 28 days of incubation at 35, 37, 39 and 42 °C (Yarrow, 1998). The experiments were conducted twice.

### Cell elongation measurements

Broth or agar of YPD, SLAD or SCLD were inoculated with overnight culture (24 h at 30 °C) and incubated for 24 h (YPD broth), 48 h (SLAD and SCLD broth) or 4 days (YPD, SLAD and SCLD agar) at 30 °C. Cells from the edges of the colonies were suspended in Milli-Q water before the cell elongation measurements. The cells were examined using a Zeiss Axiovert 135TV (Carl Zeiss) microscope equipped with a × 63 C-apochromat water-immersion objective. Images were captured by a Coolsnap™fx (RS Photometrics) camera. metamorph (Universal Imaging Corp.) image-acquisition software was used to manually obtain major and minor axis lengths of 50 cells of each strain. Cell elongation was calculated as a mean value of ratios between major and minor axis length. The experiment was conducted twice. Significance was determined using the two-tailed Student's *t*-test and *P* values <0.05 were considered significant.

### Pseudohyphal growth

Strains were propagated overnight in YPD at 30 °C and streaked onto YPD, SLAD and SCLD agar. After 1 week of incubation at 30 °C, colonies were examined for pseudohyphal growth by bright-field microscopy using a Zeiss Axioscop 50 microscope (Carl Zeiss, Germany) equipped with a × 100 objective. Images were captured by a CoolSnap™cf camera (RS Photometrics) (Lo & Dranginis, 1998). The experiment was conducted twice.

### Invasive growth assay

Strains were propagated in YPD broth overnight at 30 °C, streaked onto agar of YPD, SLAD or SCLD and incubated for 3 days at 30 °C followed by 2 days at room temperature. Invasive cells were detected after washing the cells off the agar with a gentle stream of Milli-Q water and a gloved finger (Lo & Dranginis, 1998). The agar plates were scanned (Powerlook 1120, UMAX Technologies Inc.) before and after washing for comparison. The experiment was conducted twice with separate propagations of the yeast cultures.

### Growth and maintenance of mammalian cell cultures

The human colon adenocarcinoma cell line Caco-2 was purchased from the Deutche Sammlung von Mikroorganism und Zellkulturen (DSMZ, Braunschweig, Germany) and grown in minimum essential medium (Earle's salt, 25 mM HEPES and GlutaMAX™, Invitrogen, Gibco, Rockville, MD) supplemented with 16.5% (v/v) foetal bovine serum (FBS, Cambrex Bio Science, Verviers, Belgium), 1% (v/v) nonessential amino acids (Invitrogen, Gibco) and 50 μg mL^−1^ gentamicin (Invitrogen, Gibco). The cells were routinely grown in 150 cm^2^ culture flasks (TPP, Trasadingen, Switzerland) at 37 °C in a humidified atmosphere of 5% CO_2_ and 95% air until a confluent monolayer was obtained. Culture medium was changed every second day.

### Adhesion capacity to mammalian cells

**Fig. 2:**
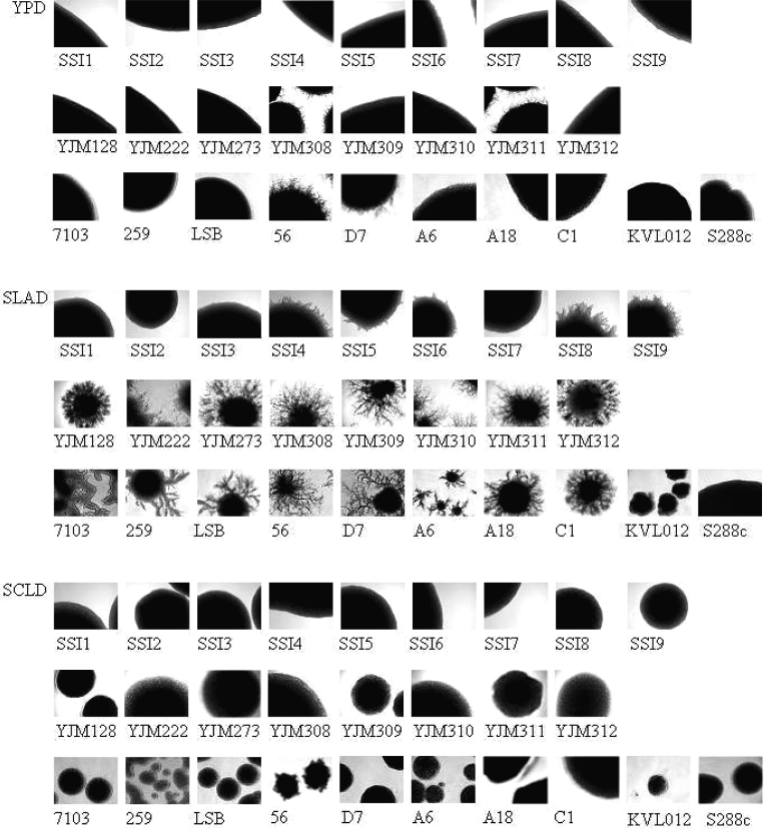
Colony morphology of *Saccharomyces cerevisiae* strains grown on solid rich-media (YPD), low-nitrogen media (SLAD) or low-carbohydrate media (SCLD).

The adhesion capacity of selected *S*. *cerevisiae* strains of clinical and nonclinical origin to Caco-2 cells was investigated *in vitro* with or without previous nitrogen starvation. The strains were selected to represent strains of both clinical and nonclinical origin. D7 is a food strain that forms extensive pseudohyphal growth on both YPD and SLAD solid media and further clustered together with the strains of clinical origin when analysed by the PFGE techniques, 7103 is a probiotic strain, SSI4 and SSI6 are strains from blood and vagina respectively, and YJM 222 (CBS 7834) and YJM 311 (CBS 7839) are strains of clinical origin having various degrees of pseudohyphal growth (Fig. 2). Additionally, YJM 222 and YJM 311 have previously been reported to have different degrees of virulence (McCusker *et al.*, 1994a). Monolayers of Caco-2 cells were seeded at a concentration of 2 × 10^5^ cells mL^−1^ and dispensed into each 400 mm^2^ well of a 12-well tissue culture plate (Nunc, Roskilde, Denmark) and incubated at 37 °C in a humidified atmosphere of 5% CO_2_ and 95% air until monolayers were developed (4 days). The yeast strains were grown overnight at 30 °C in YPD or SLAD broth or for 1 week on YPD or SLAD agar and resuspended in Caco-2 growth medium to a final concentration of 10^7^ CFU mL^−1^. The yeast suspension (1 mL) was added to each well of the tissue culture plate containing monolayers of Caco-2 cells. After 1 h of incubation the monolayers were washed three times with Caco-2 cell growth medium in order to remove nonadherent yeast. The Caco-2 cells were lysed by the addition of 0.1% (v/v) Triton-X100 (Merck), and the number of viable adherent yeast was determined by plating serial dilutions onto YPD agar. CFUs were enumerated after incubation for 48 h at 30 °C, and the adhesion capacity is described as the percentage of yeast adhered to Caco-2 cells compared with the total number of yeast added. Each adhesion assay was conducted twice (two different passages) with triplicate determinations.

### Effect of *S. cerevisiae* strains on TER

To obtain polarized monolayers, Caco-2 cells were seeded on Transwell® filter inserts (0.4 μm pore size, 12-mm id, Corning Incorporated, Corning, NY) placed into 12-well plates (diameter: 22.1 mm; Corning Incorporated) at a density of 1 × 10^5^ cells cm^−2^. A volume of 500 μL cell growth medium was added to the inner chamber (apical compartment) and 1500 μL to the outer chamber (basolateral compartment). Functional polarity was developed when electrical resistance between the apical and basolateral surface of the monolayers was >450 Ω cm^−2^. The same strains were included as described in the adhesion study. Strains grown overnight at 30 °C in YPD were resuspended in Caco-2 cell growth medium (10^7^ cells mL^−1^). The yeast suspension (500 μL) was added to the apical compartment and incubated at 37 °C in a humidified atmosphere of 5% CO_2_ and 95% air. The effects of the yeast strains on cell polarity were evaluated by measurement of the TER using the Millicell-ERS Electrical Resistance System (Millipore, Bedford, MA). The net value of the TER (Ω cm^−2^) was corrected for background resistance by subtracting the contribution of the cell free filter and the medium (110 Ω cm^−2^). The TER was measured before the addition of the yeast (*t* = 0) and then at various time intervals (1, 2, 3, 4, 5, 6, 7, 8 and 24 h). The TER of monolayers with Caco-2 cell culture media without added yeast represented the control for each experiment. Each assay was conducted twice (two different passages) with triplicate determinations.

## Results

### CLP

**Fig. 1:**
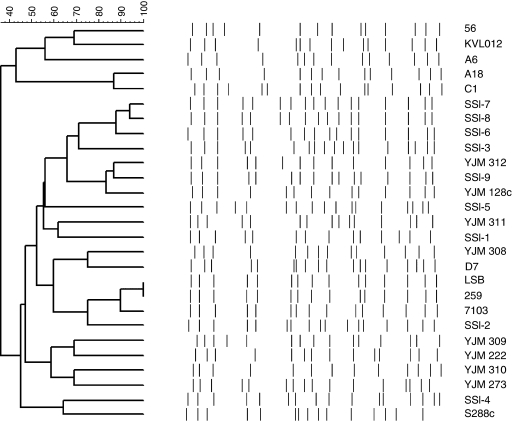
Dendrogram showing the clustering of 27 strains of *Saccharomyces cerevisiae* (three of the strains are *S. cerevisiae* var. *boulardii*, i.e. LSB, 259 and 7103) based on their CLP as determined by PFGE and evaluated using the Dice coefficient and the unweighted pair group algorithm with arithmetic averages (UPMGA).

All investigated strains had chromosome profiles typical of *S. cerevisiae* with chromosomes ranging in size from 200 to 1900 kbp (results not shown). CLP was observed among the strains, and the technique was able to separate nearly all strains. The cluster analysis is seen in Fig. 1. Five (i.e. 56, KVL012, A6, A18 and C1) of the six food strains were found to cluster together and could be separated from the remaining strains at a level of 36% similarity. The remaining food strain (D7) was found to cluster within the strains of clinical origin. Two of the probiotic strains of *S. cerevisiae* var. *boulardii* (LSB and 259) had identical profiles and clustered together with the third probiotic strain (7103) at a similarity of more than 90%. The cluster containing the probiotic strains clustered within the larger cluster of clinical strains. Also, two of the clinical strains (SSI7 and SSI8; both vaginal isolates) were quite alike and clustered together at a similarity of *c*. 90%. The five food strains (i.e. 56, KVL012, A6, A18 and C1) that clustered together were separated from the remaining strains due to a lower band size, especially of chromosome XVI and XIII (*c*. 915–945 kb) and chromosome III (*c*. 375 kb). For the haploid strain S288C, only 15 chromosomal bands were observed due to lack of separation between chromosome VII (*c*. 1120 kb) and chromosome XV (*c*. 1100 kb).

### Growth at elevated temperatures

The ability to grow at elevated temperatures was investigated in liquid media (results not shown). All investigated strains, except KVL012, were able to grow at both 35 and 37 °C. KVL012 is a domesticated ale yeast strain that has been used in the brewing industry for decades, where the temperature hardly exceeds 25 °C, and it is therefore not surprising that it does not grow at 35 °C. At 39 °C, all strains, except SSI2 and KVL012, were able to grow within 7 days. After 4 weeks of incubation at 39 °C, extensive growth was observed for SSI2. None of the investigated strains were able to grow in liquid medium at 42 °C, not even after 4 weeks of incubation.

### Cell elongation

The morphological differences of cells grown in liquid rich YPD medium and both in liquid and on solid low-nitrogen (SLAD) and low-carbon (SCLD) media were investigated (results not shown). Significantly elongated cells were found on solid SLAD compared with solid SCLD (*P* = 3 × 10^−5^). Similarly, significant elongated cells were observed when solid SLAD was compared with liquid SLAD (*P* = 0.003). Increased elongation was especially observed for four of the strains of clinical origin (SSI5, SSI7, YJM222 and YJM311) and for one of the food strains (D7). Otherwise no significant differences were observed among the media and the strains included.

### Pseudohyphal growth and invasiveness

Pseudohyphal growth was examined (Fig. 2 and Table 2). Great variations were observed among both the strains and the media used. On YPD solid medium 12% of the strains of clinical origin, none of the probiotic strains and 33% of the food strains showed pseudohyphal growth after 7 days of incubation. In general, incubation on solid SLAD (low-nitrogen medium) caused a significant increase in pseudohyphal growth. On this medium, 65% of the strains of clinical origin, 100% of the probiotic strains and 83% of the food strains showed pseudohyphal growth. On the contrary, none of the investigated strains had the ability to form pseudohyphal growth on solid SCLD (low-carbon medium). The ability to form pseudohyphal growth was not always associated with cell elongation (results not shown). As expected, the haploid strain S288C was not able to form pseudohyphal growth on any of the three media. The invasiveness of the strains at 30 °C on solid YPD, SLAD and SCLD media is additionally shown in Table 2. Variations in the invasiveness were observed among both the strains and the media used. None of the three probiotic strains showed invasiveness on any of the three media. For solid YPD (rich medium), 35% of the strains of clinical origin, none of the probiotic strains and 50% of the food strains showed invasiveness. On the solid SLAD (low-nitrogen medium), 65% of the strains of clinical origin, none of the probiotic strains and 100% of the food strains showed invasiveness. On the solid SCLD (low-carbon medium), invasiveness was seen for 24% of the strains of clinical origin, none of the probiotic strains and 67% of the food strains. Some strains did show a high degree of invasiveness on all media. This accounts for the clinical strains YJM128, YJM273, YJM309 and YJM311 as well as for the food strains D7 and 56. Both of the high invasive food strains (D7 and 56) are used in solid-state fermentation (blue veined cheeses), where their ability to invade the substrate is of importance for their establishment. In conclusion, pseudohyphal growth and invasiveness were not tightly correlated, and pseudohyphal formation appeared not always to be a prerequisite for invasiveness. No differences were observed between strains of clinical and nonclinical origin.

**Table 2:** Pseudohyphal formation and invasiveness by *Saccharomyces cerevisiae* strains when grown on solid YPD, SLAD or SCLD media at 30°C

	YPD*		SLAD*		SCLD*	
Strain/Condition	PH	IG	PH	IG	PH	IG
Strains of clinical origin						
SSI1	−	−	−	−	−	−
SSI2	−	−	−	−	−	−
SSI3	−	−	−	+	−	−
SSI4	−	−	+	++	−	−
SSI5	−	−	−	+	−	−
SSI6	−	−	−	−	−	−
SSI7	−	−	−	−	−	−
SSI8	−	−	+	−	−	−
SSI9	−	−	+	++	−	−
YJM128	−	++	+	++	−	+++
YJM222	−	−	+	−	−	−
YJM273	−	++	+	++	−	++
YJM308	+	++	+	+++	−	−
YJM309	−	++	+	++	−	+++
YJM310	−	+	+	+++	−	−
YJM311	+	+++	+	++	−	+++
YJM312	−	−	+	+	−	−
Probiotic strains						
7103	−	−	+	−	−	−
259	−	−	+	−	−	−
LSB	−	−	+	−	−	−
Food strains						
D7	+	++	+	++	−	++
56	+	+++	+	+++	−	+++
C1	−	−	+	++	−	−
A18	−	−	+	++	−	+++
A6	−	−	+	+	−	++
KVL012	−	+	−	++	−	−
Laboratory haploid strain						
S288C	−	−	−	−	−	−

* The strains were grown on solid rich (YPD), low-nitrogen (SLAD) or low-carbohydrate (SCLD) media.

The presence of pseudohyphal growth (PH) is denoted: +. For invasive growth (IG) the patterns are divided into four groups according to the intensity: −, no invasive growth; +, moderate invasive growth; ++, intermediate invasive growth; +++, enhanced invasive growth.

### Adhesion to mammalian cell cultures

**Fig. 3:**
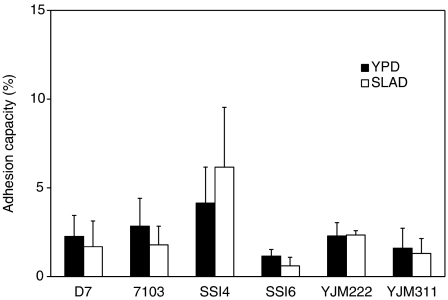
The adhesion capacity of *Saccharomyces cerevisiae* strains propagated in rich media (YPD) or low-nitrogen media (SLAD) to Caco-2 cells expressed as the percentage of adhered yeast cells (CFU mL^−1^) compared with added yeast cells (1 × 10^7^ CFU mL^−1^). Each adhesion assay was conducted twice (two different passages) with triplicate determinations. The error bars indicate the SDs.

As seen in Fig. 3, all the investigated strains adhered poorly (0.6–6.2%) after growth in liquid YPD or SLAD, and no significant difference was observed between the two media. Furthermore, no significant difference in the adhesion capacity was observed between strains of clinical and nonclinical origin. For cells of *S. cerevisiae* D7, pregrowth on solid YPD or solid SLAD did not influence the adhesion capacity of the cells. The adhesion capacity to Caco-2 cells was 2.3±1.2% after growth in liquid YPD compared with 2.5±0.4% after growth on solid YPD. Similarly, an adhesion capacity of 1.7±1.4% was observed for D7 after growth in liquid SLAD compared with 2.7±1.3% after growth on solid SLAD. Based on these results, the adhesion capacity of the yeast to human intestinal cells appears not to be influenced by the investigated pregrowth conditions (substrate consistency and nitrogen availability).

### TER

**Fig. 4:**
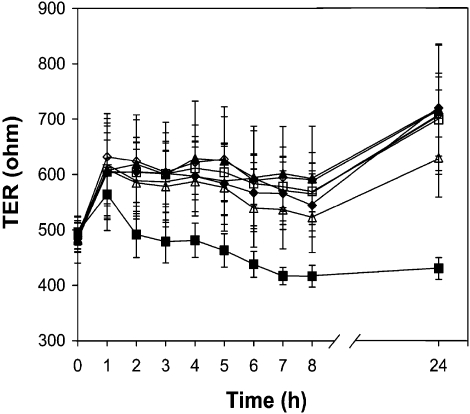
TER of polarized Caco-2 monolayers exposed to *Saccharomyces cerevisiae* of nonclinical origin [D7 (□) and 7103 (▴)] or *S. cerevisiae* strains of clinical origin [SSI4 (Δ), SSI6 (⋄), YJM222 (♦), YJM311 (+)] at a concentration of 1 × 10^7^ yeast mL^−1^ or without yeast added (▪) (control). Each assay was conducted twice (two different passages) with triplicate determinations. The error bars indicate the SD.

Both strains of clinical (SSI4, SSI6, YJM222 and YJM311) and nonclinical (D7 and 7103) origin increased the TER of polarized Caco-2 monolayers from *c*. 500 to *c*. 600 Ω after 1 h of incubation (Fig. 4). No significant changes were observed for the next 7 h. After 24 h of incubation, an increase in TER to *c*. 700 Ω was observed for five of the strains (D7, 7103, SSI6, YJM222 and YJM311), whereas the increase for SSI4 was slightly lower. The TER of Caco-2 monolayers without added yeast varied slightly around *c*. 500 Ω during the incubation period. Based on these results, *S. cerevisiae* strains were in general found to strengthen the epithelial barrier function, and no differences were observed between strains of clinical and nonclinical origin.

## Discussion

All chromosome profiles obtained in the present study were found to be typical of *S. cerevisiae*, which confirms the identification of the strains. Besides, all strains of clinical origin were clearly separated by the PFGE technique, and determination of CLP can therefore be recommended as a useful tool for profiling and tracing clinical strains of *S. cerevisiae*. A complete separation between strains of clinical origin and food strains was not obtained even though there was a tendency for the clinical and food strains to cluster separately. The probiotic strains clustered together within the strains of clinical origin. These results are comparable to results reported in other studies using typing techniques such as restriction fragment length polymorphism of chromosomal DNA, restriction analysis of digested mtDNA and PCR amplification of delta sequences (McCullough *et al.*, 1998; de Llanos *et al.*, 2004, 2006b).

Growth at 37 °C is, according to Vaughan-Martini & Martini (1998), a variable characteristic of *S. cerevisiae*. In this study all strains of clinical origin as well as the majority of the food strains were able to grow at 39 °C when grown in liquid media. None of the investigated strains in this study were able to grow at 42 °C. The present study does show that growth at 42 °C is not a prerequisite for host persistence as reported earlier (McCusker *et al.*, 1994a, b) for growth on solid media.

Both pseudohyphal growth and invasive growth were highly strain specific even though not always linked. Both characteristics were increased when grown on SLAD. This is in agreement with several other authors (Lambrechts *et al.*, 1996; Prinz *et al.*, 2004; Casalone *et al.*, 2005; Kleinschmidt *et al.*, 2005). The ratio of strains capable of invading the SLAD agar was in the same order as reported previously for wine and must strains by Casalone *et al.* (2005), who also, as in the present study, found that a few of the strains were able to invade the SLAD agar even though pseudohyphal growth was not observed. The ratio of strains capable of agar invasion was comparable for strains of clinical and nonclinical origin, and penetration of solid standardized laboratory media therefore does not appear to be linked to the ability of the strains to cause human infections. However, it cannot be excluded that factors in the gastrointestinal tract may trigger pseudohyphal growth and invasiveness as previously reported for *Candida albicans* when exposed to an undefined serum factor (Feng *et al.*, 1999).

Generally, no significant difference in adhesion to mammalian epithelial cells was found between strains of clinical and nonclinical origin. Compared with previous adhesion studies, using a porcine intestinal cell line (van der Aa Kühle *et al.*, 2005), the yeast strains adhered poorly to the human intestinal cell line, indicating a weak ability to establish in the human intestine. In the current study, we demonstrated that the TER of polarized Caco-2 monolayers increased following exposure to *S. cerevisiae* strains of both clinical and nonclinical origin, thereby strengthening the epithelial barrier function in a similar manner as seen previously for probiotic bacteria (Czerucka *et al.*, 2000; Resta-Lenert & Barrett, 2003; Otte & Podolsky, 2004; Klingberg *et al.*, 2005b). These results indicate that the examined yeast strains do not destabilize the epithelium when exposed to a healthy intestine. In addition, these results are in line with the fact that most infections of *S. cerevisiae* are reported in immunocompromised people (Cassone *et al.*, 2003; Riquelme *et al.*, 2003) with a disrupted barrier function due to infections or inflammatory bowel diseases (Madsen *et al.*, 2001), consequently allowing access of luminal yeast cells into the underlying tissue and bloodstream of the host.

In conclusion, it was not possible in the present study to identify a specific virulent factor that could be used to separate between *S. cerevisiae* strains of clinical and nonclinical origin in any of the physiological tests or in the *in vitro* mammalian cell model. In fact, all investigated strains were capable of increasing the TER of polarized monolayers of Caco-2 cells, indicating that these strains might have a protective impact on healthy human GI tract functions. These results imply that the examined yeast strains, even though some of them are isolated from clinical specimens, are not harmful when exposed orally to a healthy intestine. Based on the present observations, the immunological status of an exposed individual appears to be a much more significant factor in the development of infections caused by *S. cerevisiae* than the yeast strain characteristics.

## Statement

OnlineOpen articles are made available in accordance with the terms of the Creative Commons Deed, Attribution 2.5 (further details available from http://www.creativecommons.org), which allows Open Access dissemination of the article, but does not permit commercial exploitation or the creation of derivative works.
